# First Nationwide Molecular Screening Program in Spain for Patients With Advanced Breast Cancer: Results From the AGATA SOLTI-1301 Study

**DOI:** 10.3389/fonc.2021.744112

**Published:** 2021-11-04

**Authors:** Sonia Pernas, Patricia Villagrasa, Ana Vivancos, Maurizio Scaltriti, Jordi Rodón, Octavio Burgués, Paolo Nuciforo, Jordi Canes, Laia Paré, Marta Dueñas, Maria Vidal, Juan Miguel Cejalvo, Antonia Perelló, Antonio Llommbard-Cussac, Joan Dorca, Alvaro Montaño, Tomás Pascual, Mafalda Oliveira, Gloria Ribas, Inmaculada Rapado, Aleix Prat, Eva Ciruelos

**Affiliations:** ^1^ Catalan Institute of Oncology (ICO)- Institut d’Investigació Biomédica de Bellvitge (IDIBELL), L’Hospitalet de Llobregat, Barcelona, Spain; ^2^ SOLTI Breast Cancer Research Group, Barcelona, Spain; ^3^ Cancer Genomics Laboratory, Vall d’Hebron Institute of Oncology (VHIO), Barcelona, Spain; ^4^ Memorial Sloan Kettering Cancer Center, Memorial Hospital, New York, NY, United States; ^5^ Oncology Department, Monroe Dunaway (MD) Anderson Cancer Center, Houston, TX, United States; ^6^ Oncology Department, Vall d’Hebron University Hospital/Vall d’Hebron Institute of Oncology (VHIO), Barcelona, Spain; ^7^ Pathology Department, Hospital Clínico Universitario Valencia, Valencia, Spain; ^8^ Molecular Oncology Group, Vall d’Hebron Institute of Oncology (VHIO), Barcelona, Spain; ^9^ Molecular Oncology Unit, Centro de Investigaciones Energéticas, Medioambientales y Tecnológicas (CIEMAT), Madrid, Spain; ^10^ Institute of Biomedical Research, University Hospital “12 de Octubre”, Madrid, Spain; ^11^ Oncology Department, Hospital Clínic de Barcelona, Barcelona, Spain; ^12^ Translational Genomics and Targeted Therapeutics in Solid Tumors, Instituto de Investigaciones Biomédicas August Pi i Sunyer (IDIBAPS), Barcelona, Spain; ^13^ Breast Cancer Biology, Instituto de Investigación Sanitaria (INCLIVA) Biomedical Research Institute, Valencia, Spain; ^14^ Center for Biomedical Network Research on Cancer (CIBERONC), Valencia, Spain; ^15^ Oncology Department, Hospital Universitari Son Espases, Palma de Mallorca, Spain; ^16^ Oncology Department, Fundación para el Fomento de la Investigación Sanitaria y Biomédica (FISABIO) - Hospital Arnau de Vilanova, Valencia, Spain; ^17^ Oncology Department, Catalan Institute of Oncology (ICO), Girona, Spain; ^18^ Oncology Department, Hospital Virgen del Rocío, Sevilla, Spain; ^19^ School of Medicine, University of Barcelona, Barcelona, Spain; ^20^ Oncology Department, Hospital Universitario 12 de Octubre, Madrid, Spain

**Keywords:** breast cancer, molecular genetic, DNA sequence analyses, PAM50 subtype, molecular targeted therapy

## Abstract

**Background:**

The SOLTI-1301 AGATA study aimed to assess the feasibility of a multi-institutional molecular screening program to better characterize the genomic landscape of advanced breast cancer (ABC) and to facilitate patient access to matched-targeted therapies in Spain.

**Methods:**

DNA sequencing of 74 cancer-related genes was performed using FFPE tumor samples in three different laboratories with three different gene panels. A multidisciplinary advisory board prospectively recommended potential targeted treatments. The primary objective was to determine the success of matching somatic DNA alteration to an experimental drug/drug class.

**Results:**

Between September 2014 and July 2017, 305 patients with ABC from 10 institutions were enrolled. Tumor sequencing was successful in 260 (85.3%) patients. Median age was 54 (29-80); most tumors were hormone receptor-positive/HER2-negative (74%), followed by triple-negative (14.5%) and HER2-positive (11.5%). Ninety-seven (37%) tumor samples analyzed proceeded from metastatic sites. Somatic mutations were identified in 163 (62.7%) patients, mostly in *PIK3CA* (34%), *TP53* (22%), *AKT1* (5%), *ESR1* (3%), and *ERBB2* (3%) genes. Significant enrichment of *AKT1* mutation was observed in metastatic versus primary samples (9% *vs.* 2%; p=0.01). Genome-driven cancer therapy was recommended in 45% (n=116) of successfully screened patients, 11% (n=13) of whom finally received it. Among these patients, 46.2% had a PFS of ≥6 months on matched therapy.

**Conclusions:**

AGATA is the first nationwide molecular screening program carried out in Spain and we proved that implementing molecular data in the management of ABC is feasible. Although these results are promising, only 11% of the patients with genome-driven cancer therapy received it.

## Background

The identification of genetically driven tumor dependencies and the development of targeted therapies have dramatically improved the outcome of some cancer types including advanced breast cancer (ABC) (1–4). Routine genomic profiling is already being used in the clinical management of a variety of tumors for diagnosis, prognosis information and a better selection of targeted therapies (5, 6).

Breast cancer is a group of heterogeneous diseases with different clinical courses and treatment responsiveness. Comprehensive molecular profiling of breast cancers revealed that 80.4% of tumors harbor a genomic mutation in at least one of eight pathways with potential treatment implications (7). Traditional highly sensitive DNA sequencing methods focus on known mutational hot spots (8), while next-generation sequencing (NGS) technologies can detect also other clinically relevant genomic alterations (7).

As the number of targeted drugs increases and tumor genomic sequencing technologies become more available, genome-driven cancer treatment has substantially grown as a potential strategy of precision medicine (9–11), but costs and complexity of this technologies generally limit the access of this approach to just a minority of patients in the real clinical practice.

The SOLTI-1301 AGATA study (NCT02445482) was designed as a proof-of-concept project to better characterize the genomic landscape of metastatic breast cancer and to facilitate patient access to matched-targeted therapies into clinical trials in Spain.

## Methods

### Study Design and Patients

AGATA SOLTI-1301 was a prospective, multicenter, pilot study that tested the feasibility of implementing a molecular screening program in Spain for patients with ABC patients. A total of 10 sites within the Spanish SOLTI network participated in this study, 3 of which performed molecular analysis developed in the project. Male or female patients aged ≥18 years were eligible if they had pathologically confirmed ABC (locally advanced or metastatic), with responding, stable or progressive disease at inclusion, and had available formalin-fixed paraffin-embedded (FFPE) tumor tissue. Tumor samples could be archival or fresh from either a primary or a metastatic site. Patients could be at any point in the treatment of the metastatic disease: about to start, be receiving or had completed treatment, in any line of therapy, either within a clinical trial or according to the healthcare framework. Additional eligibility criteria included Eastern Cooperative Oncology Group (ECOG) performance status of 0-2, and a minimum predicted life expectancy of 6 months. Exclusion criteria included organic or cardiac disfunction, other types of cancer within the last 5 years, no archival tumor tissue available, or brain or bone-only metastatic disease.

All patients provided written informed consent, and the protocol was approved by the Ethics Committees from all participating institutions and Spanish Health Authorities. The study was conducted per Good Clinical Practice principles, the Declaration of Helsinki and all local regulations.

The protocol was amended on 1st November 2015 to include gene expression analyses in a retrospective manner, to provide more comprehensive and integrative molecular profiling of tumor samples. Besides, after November 2015 amendment, no bone samples were allowed due to the low yield secondary to technical issues.

Recruiting sites sent FFPE archival or fresh tumor samples to 3 different central genomic laboratories: Vall d’Hebron Institute of Oncology (VHIO) - Barcelona, Instituto de Investigación Hospital 12 de Octubre (IMAS12) – Madrid, Medical Oncology and Hematology Laboratory (INCLIVA) - Valencia, according to geographical proximity. Each of the genomic laboratories used a different customized sequencing gene panel, as per clinical practice (see Supplementary Methods and Genomic Analyses Section below). Tumor cellularity was assessed upon arrival at each institution on a hematoxylin and eosin slide before molecular analysis in each central laboratory. Samples containing at least 20-30% of tumor cells were considered suitable for DNA extraction and genomic testing. Macrodissection was performed when recommended by the local pathologist.

Results from tumoral DNA sequencing were evaluated by a molecular advisory tumor board (MAB) comprised of medical oncologists, pathologists, and molecular biologists. The MAB meetings were held every 6 weeks or as soon as 20 cases were available, whichever came first. The MAB discussed the clinical and treatment implications of the molecular abnormalities that were identified. If an actionable molecular alteration was found, meaning that it was considered potentially responsive to targeted therapy, the MAB reviewed the anonymized medical history of the patient, in collaboration with each treating physician, and proposed matched targeted therapies. Most appropriate ongoing clinical trials for each situation were reviewed and recommended in a nation-wide network.

Patients were followed up for survival until death or loss of follow-up.

### Genomic Analyses

Tumor molecular profiling by targeted NGS was performed in 3 different genomics laboratories/facilities: 1) Cancer Genomics Core at (VHIO), on the MiSeq sequencer (Illumina) covering hotspot regions of 61 genes; 2) Genomics Laboratory - (IMAS12), on the Ion Torrent (Thermo Fisher Scientific) covering 35 oncogenes, and in 3)– INCLIVA, on the Sequenom MassARRAY (Sequenom San Diego) covering 24 oncogenes. The three panels encompassed 74 different cancer-related genes. However, only 7 genes (AKT1, BRAF, EGFR, ERBB2, KIT, KRAS, PIK3CA) were common in all three panels thus not all the genes were analyzed in all the samples (**Supplemental Table S1**).

Methods for DNA extraction, quality assessment and NGS analysis performed in each of the genomics centers can be found in **Supplementary Methods**.

### *In Silico* Datasets

Publicly available breast datasets from The Cancer Genome atlas (TCGA) and the Memorial Sloan Kettering Cancer Center (MSKCC) were interrogated from cBio Cancer Genomics Portal (http://cbioportal.org).

### Gene Expression Analysis

Methods for RNA extraction, quality assessment and gene expression analysis can be found in **Supplementary Methods**. Intrinsic molecular subtypes were identified using the research-based PAM50 predictor as previously described (12, 13). Each tumor was classified into one of the following groups: Luminal A, Luminal B, HER2-enriched, Basal-like and Normal-like. PAM50 subtyping was done at the Translational Genomics and Targeted Therapeutics in Solid Tumors at IDIBAPS-Barcelona. Raw gene expression data can be found in GSE182852.

### Endpoints and Statistical Considerations

The primary objective was to assess the program’s effectiveness in assigning patients to clinical trials with targeted therapies based on their genomic tumor profile, measured as the proportion of patients assigned to selected clinical trials. Secondary objectives included the distribution of somatic mutations, the PAM50 intrinsic subtypes, the efficacy of targeted therapies based on MAB recommendations and the determination of the logistic feasibility of the program.

Sample size determination was exploratory and based on estimated recruitment at a selected time point. Assuming a technical failure rate of 15%, the study required a sample size of 305 patients to enroll 260 patients in 24 months.

Progression-free survival on matched treatment (PFS) was defined by RECIST 1.1 as the time from study treatment initiation until disease progression or death from any cause, whichever occurred first. Progression-free survival on prior therapy (pre-PFS) was defined as the time from the start of the last previous treatment to disease progression defined by RECIST 1.1 or clinical progression.

Data were summarized by frequency (%) for categorical variables and by median (range) for continuous variables. The association between two variables was evaluated using Student’s t-test, Pearson’s χ2 test or Fisher’s exact test. The values of p have been considered at a descriptive level only. All P values were two-sided with an α of 0.05. Statistical analyses were conducted using R version 3.5.1 and AutoDiscovery 3.1 (Butler Scientifics, Barcelona, Spain).

## Results

### Patient’s and Tumor’s Characteristics

**Figure 1:**
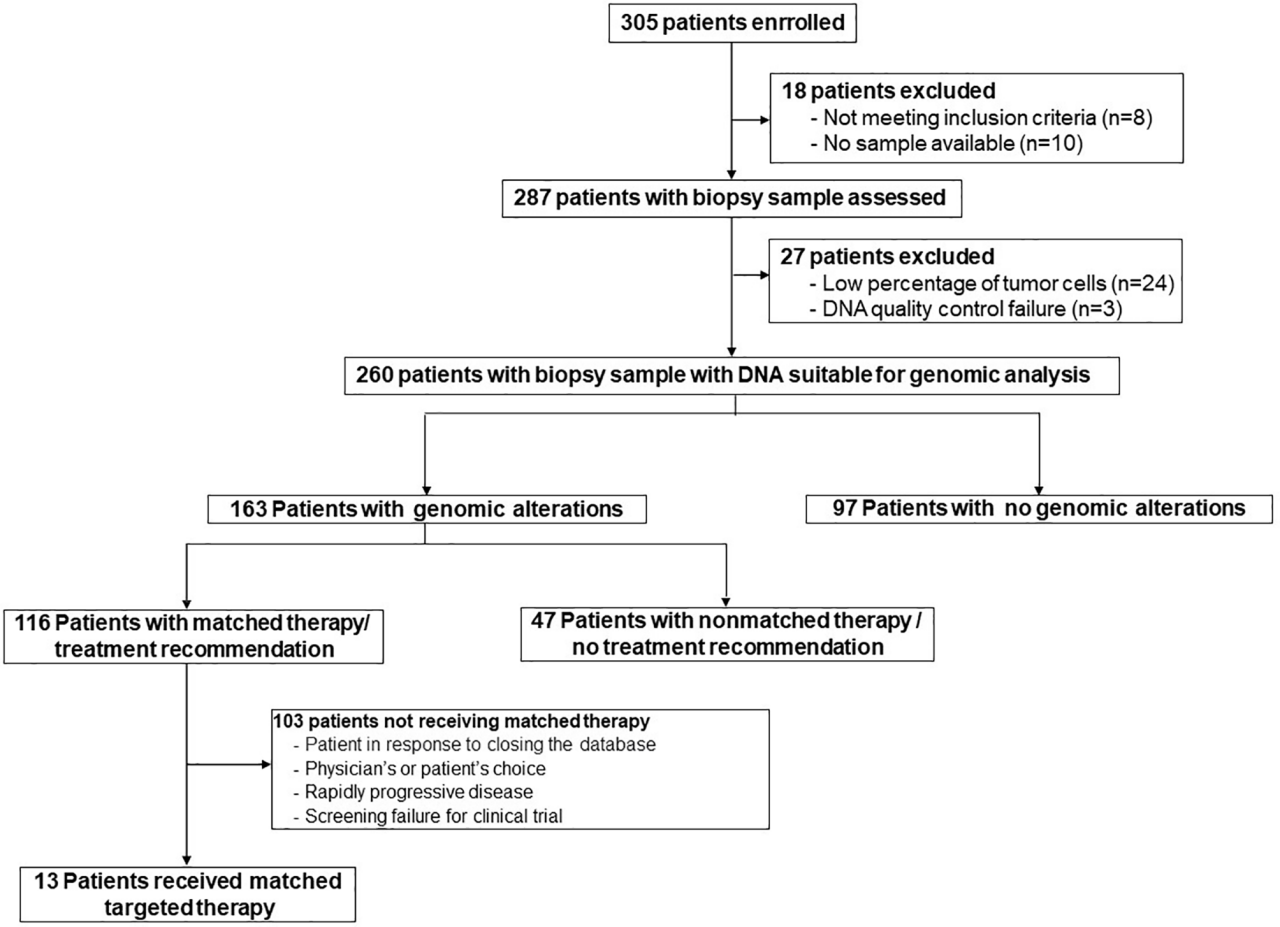
Flow chart of the SOLTI-1301 AGATA study.

Between September 2014 and July 2017, 305 patients with ABC were assessed for eligibility across 10 sites in Spain. Eighteen patients were excluded due to the lack of tumor samples available or not meeting inclusion criteria (**Figure 1**). Genomic analyses were successfully performed in 260 out of the 287 (85.2%) patients. The causes for tumor sample failure exclusions were a low percentage of cancer cells (n=24) and poor quality of the DNA (n=3). Patient characteristics are listed in **Table 1**. The median age was 54 years, 95.4% had ECOG PS 0 or 1, 79.6% had received adjuvant therapy and over 76.2% of patients had received 2 or more prior therapy lines in the metastatic setting. Among the 260 tumor samples profiled, 163 (62.7%) were obtained from primary tumors and 97 (37.3%) from diverse metastatic sites: liver (25%), lymph node (23%), breast (11%), bone (9%), ovary (8%), skin (7%), chest wall (6%), lung (6%), brain (3%) and others (2%).

**Table 1:** Patients characteristics.

Evaluable patients (n = 260)	
**Sex**
Female	259 (99.6%)
Male	1 (0.4%)
**Age (years) at inclusion**
Median (range)	54 (29-80)
<50	95 (36.5%)
≥50	165 (63.5%)
**Histological type**
Ductal Carcinoma	227 (87.3%)
Lobular Carcinoma	19 (7.3%)
Medular Carcinoma	2 (0.8%)
Papillary Carcinoma	2 (0.8%)
Others	8 (3.8%)
**ECOG performance status**
0	120 (46.2%)
1	128 (49.2%)
2	6 (2.3%)
**Immunohistochemistry receptor status**
Triple Negative	46 (17.7%)
HR-negative/HER2-positive	8 (3.1%)
HR-positive/HER2-positive	22 (8.5%)
HR-positive/HER2-negative	184 (70.8%)
**Therapy in early disease**
Neo/adjuvant chemotherapy	194 (74.6%)
Neo/Adjuvant endocrine therapy	144 (55.4%)
Neo/Adjuvant anti HER2 therapy	19 (7.3%)
No adjuvant treatment	53 (20.4%)
**Prior lines in the metastatic setting at inclusion**
Median (min-max)	3 (0-18)
0	12 (4.6%)
1	50 (19.2%)
2-3	78 (30%)
>3	120 (46.2%)

### Comprehensive Molecular Profile

Among all evaluated patients (n=260), at least one somatic mutation was identified in 163 patients (62.7%) and 46 out of these 163 (28.2%) had multiple genomic alterations with an average of 1.4 mutations per patient (**Supplemental Table S2**). No mutation was detected in 97 (37.3%) patients.

**Figure 2:**
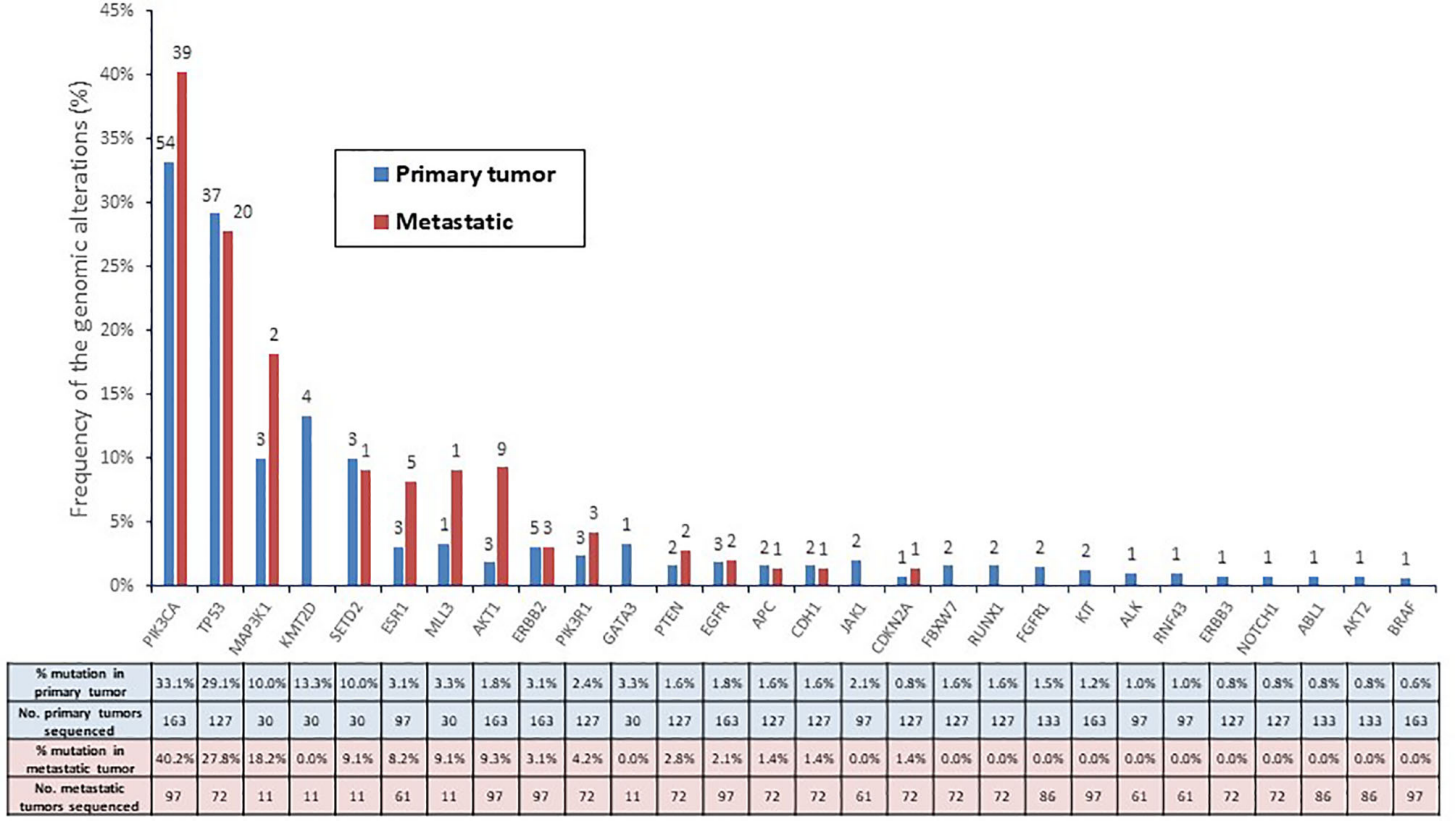
Frequency (percentage) of the genomic alterations in all patients profiled according to primary (n = 163) and metastatic tumors (n = 97).

Considering the number of tumors analyzed for each alteration, most frequent alterations per patient were detected in PIK3CA (34%, n=260), TP53 (28%, n=199), AKT1 (5%, n=260), ESR1 (5%, n=158), and ERBB2 (3%, n=260). Based on tumor site, the most frequent mutations detected in primary tumors were: PIK3CA (33.1%, n=163), TP53 (29.1%, n=127) and KMT2D (13.3%, n=30). The genomic profile from the metastatic sites showed enrichment in some mutations as compared with primary tissue, such as PIK3CA (40.2% *vs.* 33.1%, p=NS) and AKT1 (9.3% *vs.* 1.8%, p=0.01) (**Figure 2**). Among all mutations detected, 74% were potentially actionable (74.3% in primary tissue and 78.6% in metastatic tissue).

We performed a comparison of the proportion of primary tumors with gene alterations between our study and the TCGA dataset (**Supplemental Table S3**). 74 of the 76 cancer-driven genes did not show a significant difference in the proportion of mutated tumors between both datasets. The only two genes with a significant difference in the proportion of mutated tumors between both datasets were ESR1 (3.1% *vs.* 0.4%; p =0.007) and CDH1 (1.6% *vs.* 6.7%; p=0.007).

To further explore the differences in the mutation frequency among the metastatic tumor samples, we compared the data from AGATA cohort with the dataset from the Memorial Sloan Kettering Cancer Center (MSKCC). Among the patients with metastatic breast cancer included in the MSKCC dataset, 73 of the 76 cancer-driven genes did not show a significant difference in the proportion of mutated tumors between both datasets (**Supplemental Table S4**). The only three genes with a significant difference in the proportion of mutated tumors between both datasets were AKT1 (9.3% *vs.* 4.3%; p=0.049), PIK3R1 (4.2% *vs.* 1.0%; p=0.040), and CDH1 (1.4% *vs.* 13.7%; p=0.003).

### Primary and Efficacy Endpoints

The primary endpoint could be evaluated in all patients with genomic alterations detected since all cases were reviewed and interpreted by the MAB (n=163). Among these patients, the MAB was able to provide a treatment recommendation in 116 (71.2%). Of them, 13 patients (11.2%) were eventually treated with a matched-targeted therapy, either as single-agent or in combination with other drug (**Figure 1**). Overall, considering the total number of patients with suitable DNA for genomic analyses (n=260), 13 (5%) were eventually treated with a matched-targeted therapy. The MAB also recommended potential clinical trials based on real-time availability. Results from additional H&E staining and gene expression were subsequently reviewed by the MAB to reinforce or to add value to the treatment recommendation.

**Figure 3:**
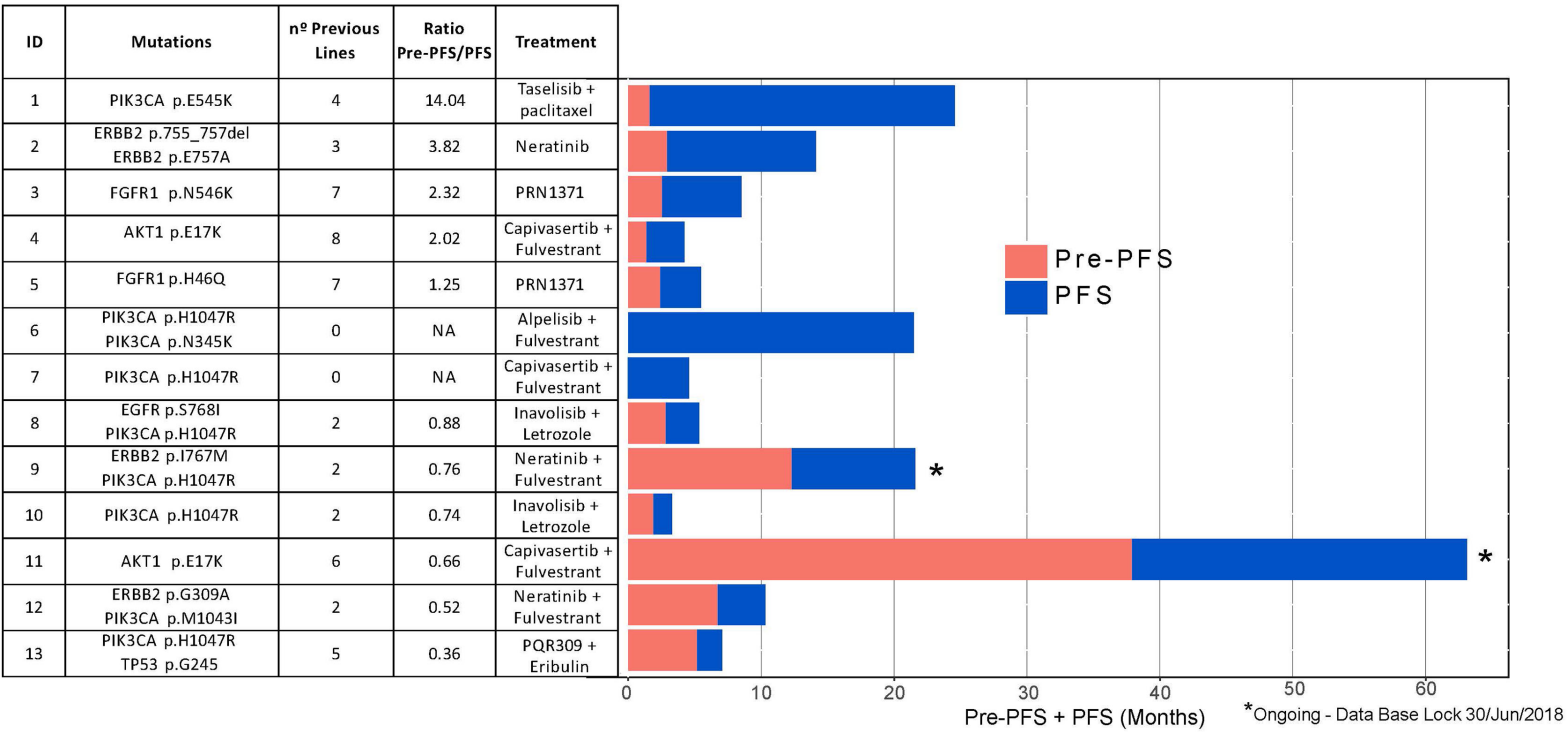
Individual progression-free survival 1 (PFS1) and progression-free survival 2 (PFS2) among patients treated with genome-driven cancer therapy (n = 13).

Genomic alterations among the 13 patients who received matched-targeted treatment included ERBB2 mutation (n=3), FGFR1 mutation (n=2), AKT1 mutation (n=2) and PIK3CA alteration (n=8) (**Figure 3**).

The group of patients with MAB recommendations (n=116) and the nonmatched therapy (n=47) group had similar characteristics except for the hormone receptor (HR)-status: nonmatched therapy group included more patients with HR-negative tumors (36.2% *vs.* 9%) (**Supplemental Table S5**). The median number of prior therapies in the metastatic setting was 3 in both groups. **Supplemental Table S6** summarizes the therapeutic recommendations made by the MAB according to the targeted mutations.

Among the 13 patients treated with genome-driven cancer therapy, 46.2% had a PFS of ≥6 months on matched therapy. The progression-free survival ratio (PFS/Pre-PFS1) derived from genome-driven cancer therapy when compared to the last therapy received is reported in **Figure 3**. In 5 out of 11 patients (45.5%), this ratio was favorable to targeted therapy. Importantly, two patients were still on treatment under the recommended targeted therapy (at the time of the database lock).

### PAM50 Intrinsic Subtype Distribution

**Figure 4:**
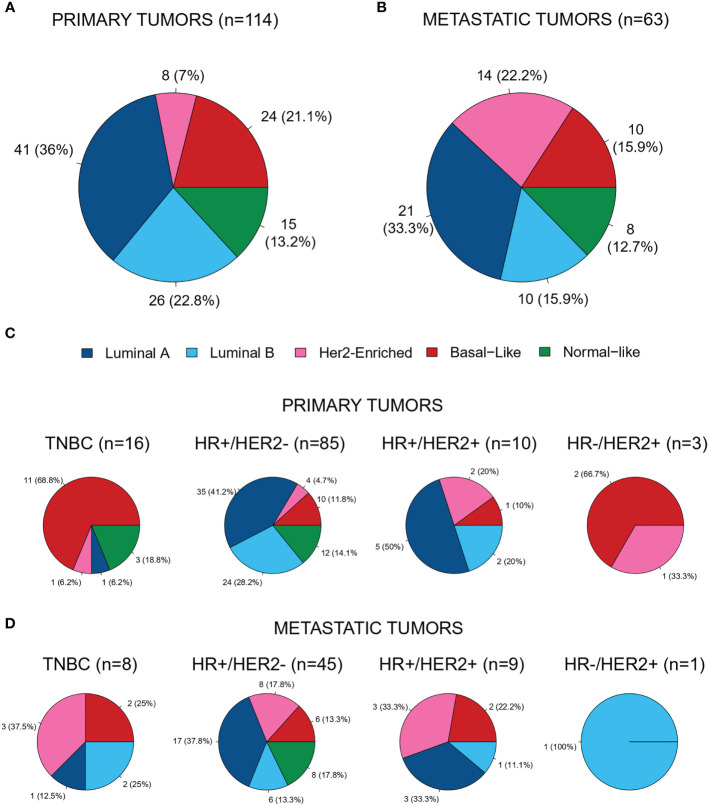
Distribution of the PAM50 intrinsic subtypes in **(A)** primary tumor samples (n = 114; 64.4%) **(B)** metastatic samples (n = 63; 35.6%) **(C)** in primary tumors across immunohistochemistry subtypes and **(D)** in metastatic tumors across immunohistochemistry subtypes.

As part of the comprehensive molecular analysis planned in this study, gene expression was performed in 177 out of the 260 samples (68%). Intrinsic subtype distribution (n=177 samples) was as follows: 34% Luminal A (n=62); 21% Luminal B (n=36); 13% HER2-enriched (n=22); 19% Basal-like (n=34) and 13% Normal-like (n=23). Among these samples, 114 (64.4%) were from primary tumors and 63 (35.6%) were from metastatic sites. The distribution of the PAM50 intrinsic subtype classification differed between primary (**Figures 4A, C**) and metastatic tumors (**Figures 4B, D**): HER2-Enriched subtype was significantly more frequent in the metastatic tissue (22% *vs.* 7%; p=0.005).

**Figure 5:**
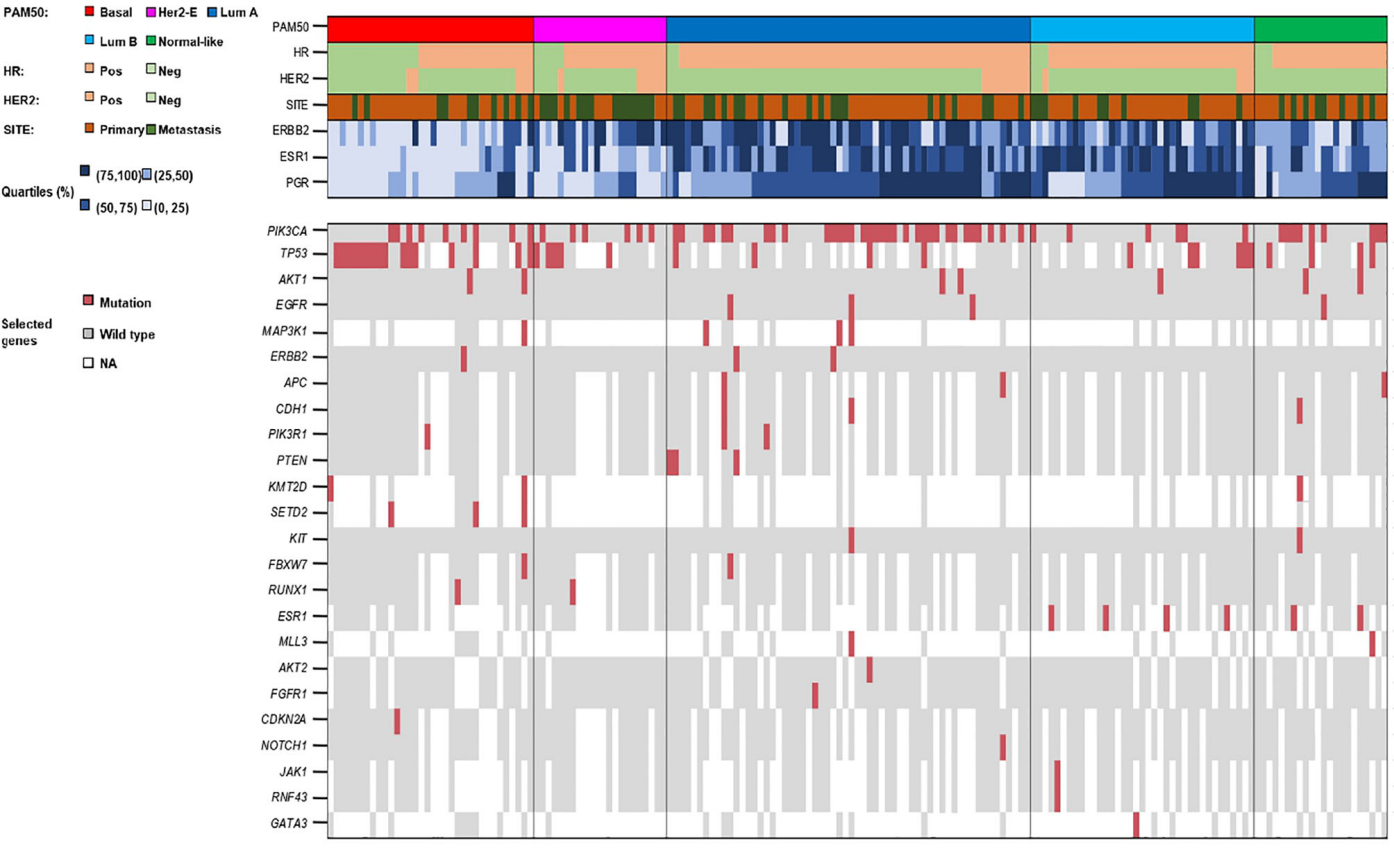
Summary of biological and genomic features of the 177 gene expression profiled tumors.

**Figure 6:**
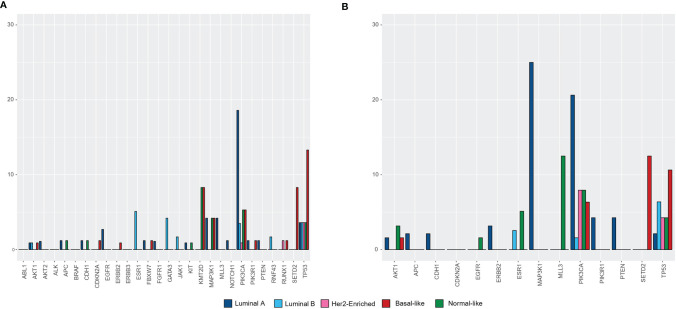
Percentage of patients with genomic alterations based on the PAM50 intrinsic subtype in **(A)** primary tumors (n = 114), **(B)** in metastatic tumors (n = 63).

We also analyzed the distribution of genomic alterations based on the PAM50 intrinsic subtype and tumor sample origin (**Figures 5**, **6**). In the luminal A subtype, the most common mutation identified in primary tumors was in PIK3CA (21/113, 18.6%), whereas MAP3K1 was the most frequently found in metastatic samples (2/8, 25.0%). TP53 mutation was mostly identified in Basal-like subtype both in primary (11/83, 13.2%) and metastatic (5/47, 10.6%) tissue.

### Program Feasibility

The whole process between the first patient’s sample shipment to the lab and the MAB recommendation took a median of 64 days (range 15-459). Median turnaround time to obtain sequencing results was 14 days (3-424).

The most common causes for not getting genome-driven cancer treatment in mutated patients (n=103) were: no clinical trials available according to the MAB recommendation at that time (n=47), no progressive disease at the time of reporting results (n=13), not meeting the inclusion/exclusion criteria for recommended clinical trials (n=12), rapidly progressive disease (n=9), and physician’s or patient’s opinion (n=7).

## Discussion

The AGATA SOLTI-1301 was a pilot study to implement a nationwide molecular screening platform for ABC patients, to better characterize the genomic landscape of the disease and to facilitate patient access to matched-targeted therapies in Spain. We have shown that running a multi-center molecular screening program is feasible in a real world setting, when scientific and clinical collaborative structures exist. We have achieved the sequencing of 260 tumors from ABC patients, with a screening failure rate of 15% due to technical issues, a similar value to that obtained in any clinical trial (14). From all evaluated patients, at least one somatic mutation was identified in 63%, and 28% of these patients had multiple targetable genomic alterations. Moreover, in about half of the patients a treatment recommendation according to their tumor profile was proposed, even though just 11% of them were eventually treated accordingly, mainly on a clinical trial.

The effectiveness of this pilot study to assign patients into genome matched clinical trials has been slightly lower than other reported series (15–17). In the MOSCATO-01 trial (15), a French unicentric, pan tumor screening program, 49% of patients had actionable mutations and therefore a targeted treatment recommendation could be offered, similarly to what we have observed in AGATA. However, 19% of the screened patients in that trial received a targeted therapy. Another example is the Safir01/UNICANCER trial (17), a metastatic BC multicenter study focused on PIK3CA and AKT1 pathways. In this study, a targetable alteration was identified in 46% of patients (15). However, only 13% of patients could finally be treated with targeted therapy. The authors state that the lack of access to drugs for patients may lead to this lower percentage, an explanation that we have also observed in our study. In the AGATA SOLTI-1301 study, the main reasons for not receiving the recommended targeted therapy were related to physician’s or patient’s decision, screening failure from clinical trials or to rapid progressive disease or death once the recommendation from the MAB was notified.

The results of this study showed that the distribution of molecular alterations differed between primary and metastatic tumor samples. From 260 analyzed tumor samples, only 37% proceeded from metastatic tissue, mainly from liver and lymph node metastases. Somatic mutations were found in 63% of these patients; 76% of those were potentially actionable. Those results are similar to those reported in larger molecular screening programs (15–17). In our study, PIK3CA and TP53 mutations were the most frequent mutations found both in primary and metastatic samples, however, AKT1 mutations were significantly more prevalent in metastatic samples (18). Our data suggest that it is preferable to match patients to potential therapies based on the analysis of their metastatic tumor, rather than archival material of their primary tumor, as metastatic tumors often develop new mutations during the metastatic process and treatment course (19). This finding reinforces the need to perform biopsies at metastatic sites, whenever is feasible.

Among the 13 patients treated with genome-driven cancer therapy, PFS/Pre-PFS ratio was favorable to targeted therapy in 5 out of 11 patients (45.5%) even though they had received 3 or more previous lines of treatment for metastatic disease. Two patients were still on treatment at the time of the database lock. Our results corroborate that matched therapy is a valid strategy, but we are far from hitting the ideal treatment if there are few one alteration available with a described clinical utility to address. More efforts should be made to have all available molecular information integrated when selecting treatment.

Another finding in our study is that PAM50 HER2-enriched subtype was more prevalent in metastatic tissue than in primary tumors. This subtype is characterized by having a more aggressive profile, as described in previous studies by our group (20–22). New treatment strategies for patients based on tumor molecular intrinsic subtype in the metastatic setting should be explored. This warrants molecular pre-screenings implementation in clinical trials, and identification of the molecular alteration of interest early on the natural history of patient’s disease. In line with this strategy, SOLTI is performing several trials where patients are screened and treated according to their intrinsic subtype profile (NCT04142060, NCT02448420, NCT04251169).

Our study has several limitations. First, three different genomic laboratories each with a different customized panel based on a different technology were used, and this could limit the robustness of the results. Second, most of the study population was heavily pretreated, limiting the possibility that the patients could be enrolled in clinical trials with targeted therapies either because of quick worsening of patient’s condition or even death. Third, none of the panels used in this study included genes associated with familial breast cancer nor did they determine copy number alterations, limiting potential targeted therapies that could be recommended such as PARP inhibitors or HER2 targeted therapies Fourth, no blood samples were collected at the time of the study completion, so no match plasma *vs.* tissue results could be analyzed in order to test liquid biopsy utility in our series (23). Finally, the turnaround time for the molecular report, as well as the time elapsed between study inclusion and the MAB report may have influenced the clinical utility of the results.

Remarkably, our study was designed to represent the reality of Spanish healthcare system considering different genomic assays between centers, different matched clinical trials and targeted therapies access in across different regions of the country. AGATA SOLTI-1301 study differs in that way from other molecular screening programs with customized panels and a more selected population, reflecting the real-world scenario. It should be noted that the different customized panels used in our study, with different number of genes analyzed (from 24 to 61 genes per sample), could limit the possibility of finding targeted therapies against other alterations. Rather, it is a situation that reflects routine clinical practice, where sequencing panels with large numbers of genes are limited mainly for cost-effective reasons, and where custom or commercial panels with fewer than 100 genes are commonly used. Moreover, all the molecular results were reviewed by a multidisciplinary expert committee that provided a therapeutic recommendation for each patient, rigorously translating data from molecular profiling into a treatment recommendation by an expert consensus. This is a relevant fact given the increase of companies that offer tumor DNA-seq services directly to patients without evaluating this information with clinical data and previously received treatments (24).

Efforts should be made to learn more about the clinical implications of differences at gene expression level and tumor protein values to integrate it with genomic mutations. Additionally, systems biology approach integrating all the information obtained at the DNA, RNA, DNA methylation and protein level is essential to develop and validate biomarker assays.

Following the steps of AGATA SOLTI-1301study, one strategy to overcome the barriers of implementing NGS in the clinic may be to promote the active participation of metastatic BC patients in the management of their disease. With this in mind, we designed HOPE (SOLTI-1903, NCT04497285), a national real-world study where patients lead their inclusion, participation and follow-up in the study through a digital tool that guides them in every step of the journey. Our objective in HOPE is empowering ABC patients and gather real-world data about the utilization of molecular information in the management of metastatic BC. Appropriate frameworks are needed to ensure that precision medicine can benefit a wider number of cancer patients and not just a minority.

## Conclusion

AGATA is the first molecular screening program multicenter performed in Spain. We demonstrated the feasibility of implementing tumor genomic data in the management of advanced breast cancer patients in a real world setting. Further studies are needed to evaluate if a more comprehensive and integrated molecular profiling could improve the survival outcomes of advanced breast cancer.

## Data Availability Statement

The original contributions presented in the study are included in the article/**Supplementary Materials**. Further inquiries can be directed to the corresponding author.

## Ethics Statement

The studies involving human participants were reviewed and approved by Comite Etico de Investigacion Clinica del Hospital Universitari Vall d’Hebron. The patients/participants provided their written informed consent to participate in this study.

## Author Contributions

All authors participated in the design and/or interpretation of the reported results and participated in the acquisition and/or analysis of data. In addition, all authors participated in drafting and/or revising the manuscript and provided administrative, technical or supervisory support.

## Funding

This study was supported by a grant from Novartis. This study was funded, in part, by the project PI 15/01508, integrated in the Plan Estatal I+D+I and co-funded by Instituto de Salud Carlos III - Subdirección General de Evaluación and European Regional Development Fund (ERDF) (to EC) and by a grant from Mutua Madrileña Foundation (to EC). The decisions and responsibilities of this study were all under the sponsor: SOLTI Breast Cancer Research Group. The funder was not involved in the study design, collection, analysis, interpretation of data, the writing of this article or the decision to submit it for publication.

## Conflict of Interest

SP reports advisor/consultant role for AstraZeneca, Daiichi-Sankyo, Polyphor, Novartis, SeattleGenetics, Pierre Fabre, Eisai, and Roche. Advisory role of A.P. for Nanostring Technologies, Pierre Fabre, Roche, Pfizer, Novartis, BMS and Roche. MO reports Grant/Research Support (to the Institution) from AstraZeneca, Philips Healthcare, Genentech, Roche, Novartis, Immunomedics, Seattle Genetics, GSK, Boehringer-Ingelheim, PUMA Biotechnology, and Zenith Epigenetics; personal fees from Roche, Seattle Genetics, and Novartis; consultant role for Roche/Genentech, GSK, PUMA Biotechnology, AstraZeneca, and Seattle Genetics; and travel Grants from Roche, Pierre-Fabre, Novartis, and Eisai. APr has declared personal honoraria from Pfizer, Novartis, Roche, MSD Oncology, Lilly and Daiichi Sankyo, travel, accommodations, and expenses paid by Daiichi Sankyo, research funding from Nanostring Technologies, Roche and Novartis, consulting/advisory role for Nanostring Technologies, Roche, Novartis, Pfizer, Oncolytics Biotech, Amgen, Lilly, MSD, PUMA and Daiichi Sankyo, Inc. EC reports personal fees from Pfizer and non-financial support from Pfizer during the conduct of the study; personal fees from Roche, personal fees from Lilly, personal fees from Astra Zeneca, personal fees from Novartis, and personal fees from MSD.

The remaining authors declare that the research was conducted in the absence of any commercial or financial relationships that could be construed as a potential conflict of interest.

## Publisher’s Note

All claims expressed in this article are solely those of the authors and do not necessarily represent those of their affiliated organizations, or those of the publisher, the editors and the reviewers. Any product that may be evaluated in this article, or claim that may be made by its manufacturer, is not guaranteed or endorsed by the publisher.
