# Supplementary material for: First Nationwide Molecular Screening Program in Spain for Patients With Advanced Breast Cancer: Results From the AGATA SOLTI-1301 Study
Source: Front Oncol. 2021 Nov 4;11:744112. doi: 10.3389/fonc.2021.744112 (PMC8600133; doi:10.3389/fonc.2021.744112)
Supplement: Supplementary file 1 [file DataSheet_1.docx]

**Supplemental methods**

*Target sequencing using* *Amplicon-seq VHIO-Card panel*

DNA extraction from FFPE tumor samples was performed using the Maxwell FFPE Tissue LEV DNA Purification Kit, following the manufacturer’s instructions. The minimum tumor content was set to 20%, to ensure subclonal somatic mutations identification or calling.

An initial multiplex-PCR with a proof-reading polymerase was performed on samples using a panel of over 800 primer pairs targeting frequent mutations in oncogenes and several tumor suppressors. A total of 61 genes were analyzed (**Supplemental Table S1**).

Indexed libraries were pooled and loaded onto a MiSeq instrument and sequencing performed (2X100). The initial alignment was performed with BWA after primer sequence clipping and variant calling performed with the GATK Unified Genotyper and VarScan2 followed by ANNOVAR annotation. Mutations were called at a minimum of 3% allele frequency. SNPs were filtered out with dbSNP and 1000 genome datasets (MAF>0.05). All detected variants were manually checked.

*Target gene sequencing using* *Ion torrent Research Institute 12 Octubre panel (i+12)*

DNA was extracted from FFPE tissues using QIAamp DNA FFPE Tissue Kit from Qiagen (Hilden, Germany) following the manufacturer’s instructions. The minimum tumor content was set to 20%, to ensure subclonal somatic mutations identification or calling.

Massive sequencing was carried out with Ion Torrent Proton sequencer (Thermo Fisher Scientific, Waltham, MA, USA).

Ion Ampliseq custom panel which covered the coding and splicing regions of 14 genes and the hotspot regions of 22 genes frequently mutated in breast cancer (**Supplemental table S1**). A total of 419 amplicons were generated from genomic DNA. Following the manufacturer’s protocol, sequencing libraries (each one carrying a unique barcode) were elaborated in duplicate using Ion AmpliSeq Library Kit 2.0 (Thermo Fisher Scientific). Then, a reaction filter created millions of micro reactions in microspheres in which clonal amplification occurs. The product was transferred to an Ion PI Chip v3 (Thermo Fisher Scientific) for sequencing at a target coverage of 500X. Data analyses were performed by the software Ion Reporter 4.0 (Thermo Fisher Scientific) which identified single nucleotide polymorphisms (SNPs) and indels.

*Target gene sequencing using* *Sequenom MassARRAY somatic mutation genotyping, INCLIVA*

DNA was extracted from FFPE tissues. The minimum tumor content was set to 30%, to ensure subclonal somatic mutations identification or calling.

The Sequenom MassARRAY and OncoCarta Panel v1.0 were used following the manufacturer's protocol (Sequenom, San Diego, CA, USA; <http://agenabio.com/oncocarta-panel>). The panel consisted of 24 multiplex assays capable of detecting 238 mutations in 19 oncogenes. DNA was amplified using the OncoCarta PCR primer pools. Unincorporated nucleotides were inactivated by shrimp alkaline phosphatase (SAP), and a single base extension reaction was performed using extension primers that hybridize immediately adjacent to the mutations and a custom mixture of nucleotides. Salts were removed by the addition of a cation exchange resin. Multiplexed reactions were spotted onto SpectroCHIP II arrays, and DNA fragments were resolved by MALDI-TOF on the Compact Mass Spectrometer (Sequenom, San Diego, CA).

Two additional customized mutation panels were used. These panels were designed in collaboration with the Cancer Genomics Group at the VHIO and included, in 12 multiplexes, a total of 107 somatic mutations in 15 genes. These two panels included 49 additional positions in 6 additional genes. Therefore, a total of 287 different positions in 25 oncogenes were checked (**Supplemental Table S1**).

Data were analyzed using the Sequenom MassARRAY Typer Analyser 4.0 Software to visualize the mass spectra for mutations and to determine the frequency of mutant and wild-type alleles. The lower threshold for mutation detection was set at 10%^14–16^. Mutations were manually reviewed using visual and raw spectrum patterns. Two different personnel in the laboratory scored mutations.

*Average depth*

The targeted regions were covered on average at 2,355.98 (143-17641) of sequencing deep. Nonsynonymous somatic mutations were called from the clinical cancer panels in regions covered by at least >300X sequencing depth and a fixed threshold of 2.5 to 10% variant allele fraction (VAF).

*Gene expression analysis*

A section of FFPE breast tissue was first examined with hematoxylin and eosin staining from the same core biopsy than the one used for nucleic acid extraction and molecular analysis to confirm the presence of invasive tumor cells (≥10%) and to determine the minimum tumor surface area (10 mm2). For RNA purification (High Pure FFPET RNA isolation kit, Roche, Indianapolis, IN, USA) at least two 10μm FFPE slides were used. Macrodissection was performed to avoid contamination with normal tissue. A minimum of ~125 ng of total RNA was used to measure the expression of 55 breast cancer-related genes using the nCounter platform (Nanostring Technologies, Seattle, WA, USA), including the 50 genes of the PAM50 subtype predictor, androgen receptor and 4 immune genes (*CD4, CD8, PD1* and *PDL1*). Data were normalized using 5 housekeeping genes (*ACTB, MRPL19, PSMC4, RPLP0,* and *SF3A1*), and log2 transformed (**Additional file 2**).

**Supplemental material**

**Table S1 –** Detailed gene panels used in AGATA per genomic center

**Table S2** – Number of the somatic mutation in the 260 evaluated patients

| **Number of mutations** | 0 | 1 | 2 | 3 | 4 | 5 | 6 |
| --- | --- | --- | --- | --- | --- | --- | --- |
| **Number of patients** | 97 | 117 | 32 | 8 | 2 | 3 | 1 |

**Table S3. Table with the actual proportions of mutation in cancer-driven genes in primary tumors in both AGATA and TCGA datasets.**

|  | **AGATA** | | **TCGA** | |  |
| --- | --- | --- | --- | --- | --- |
|  | **Proportion tumors wit mutation** | **N Primary tumors** | **Proportion tumors wit mutation** | **N Primary tumos** | **p-value** |
| PIK3CA | 33.1% | 163 | 35.3% | 507 | 0.608 |
| TP53 | 29.1% | 127 | 37.5% | 507 | 0.0777 |
| MAP3K1 | 10.0% | 30 | 7.7% | 507 | 0.649 |
| KMT2D | 13.3% | 30 | 7.5% | 507 | 0.2508 |
| SETD2 | 10.0% | 30 | 1.0% | 507 | 0.052 |
| ESR1 | 3.1% | 97 | 0.4% | 507 | **0.007*** |
| MLL3 | 3.3% | 30 | 7.5% | 507 | 0.390 |
| AKT1 | 1.8% | 163 | 2.4% | 507 | 0.654 |
| ERBB2 | 3.1% | 163 | 1.4% | 507 | 0.157 |
| PIK3R1 | 2.4% | 127 | 2.6% | 507 | 0.899 |
| GATA3 | 3.3% | 30 | 10.7% | 507 | 0.1952 |
| PTEN | 1.6% | 127 | 3.6% | 507 | 0.253 |
| EGFR | 1.8% | 163 | 0.8% | 507 | 0.275 |
| APC | 1.6% | 127 | 0.6% | 507 | 0.077 |
| CDH1 | 1.6% | 127 | 6.7% | 507 | **0.007*** |
| JAK1 | 2.1% | 97 | 0.4% | 507 | 0.061 |
| CDKN2A | 0.8% | 127 | 0.4% | 507 | 0.560 |
| FBXW7 | 1.6% | 127 | 0.4% | 507 | 0.761 |
| RUNX1 | 1.6% | 127 | 3.6% | 507 | 0.0765 |
| FGFR1 | 1.5% | 133 | 0.2% | 507 | 0.051 |
| KIT | 1.2% | 163 | 1.0% | 507 | 0.828 |
| ALK | 1.0% | 97 | 0.6% | 507 | 0.657 |
| RNF43 | 1.0% | 97 | 0.6% | 507 | 0.657 |
| ERBB3 | 0.8% | 127 | 1.6% | 507 | 0.4989 |
| NOTCH1 | 0.8% | 127 | 0.4% | 507 | 0.560 |
| ABL1 | 0.8% | 133 | 0.8% | 507 | 1.000 |
| AKT2 | 0.8% | 133 | 0.2% | 507 | 0.2794 |
| BRAF | 0.6% | 163 | 0.6% | 507 | 1.000 |

**Table S4. Table with the actual proportions of mutation in cancer-driven genes metastatic tumors in both AGATA and MSK datasets.**

|  | **AGATA** | | **MSK** | |  |
| --- | --- | --- | --- | --- | --- |
|  | **Proportion tumors wit mutation** | **N Metastatic tumors** | **Proportion tumors wit mutation** | **N Metastatic tumors** | **p-value** |
| PIK3CA | 40.2% | 97 | 36.20% | 395 | 0.465 |
| TP53 | 27.8% | 72 | 37.5% | 395 | 0.115 |
| MAP3K1 | 18.2% | 11 | 6.8% | 395 | 0.147 |
| KMT2D | 0.0% | 11 | 3.8% | 395 | 0.511 |
| SETD2 | 9.1% | 11 | 2.5% | 395 | 0.182 |
| ESR1 | 8.2% | 61 | 9.1% | 395 | 0.819 |
| MLL3 | 9.1% | 11 | 9.4% | 395 | 0.973 |
| AKT1 | 9.3% | 97 | 4.3% | 395 | **0.049*** |
| ERBB2 | 3.1% | 97 | 5.1% | 395 | 0.405 |
| PIK3R1 | 4.2% | 72 | 1.0% | 395 | **0.040*** |
| GATA3 | 0.0% | 11 | 16.5% | 395 | 0.142 |
| PTEN | 2.8% | 72 | 8.4% | 395 | 0.098 |
| EGFR | 2.1% | 97 | 1.3% | 395 | 0.556 |
| APC | 1.4% | 72 | 1.5% | 395 | 0.949 |
| CDH1 | 1.4% | 72 | 13.7% | 395 | **0.003*** |
| JAK1 | 0.0% | 61 | 2.0% | 395 | 0.266 |
| CDKN2A | 1.4% | 72 | 0.5% | 395 | 0.379 |
| FBXW7 | 0.0% | 72 | 1.0% | 395 | 0.395 |
| RUNX1 | 0.0% | 72 | 4.8% | 395 | 0.058 |
| FGFR1 | 0.0% | 86 | 0.8% | 395 | 0.406 |
| KIT | 0.0% | 97 | 1.3% | 395 | 0.406 |
| ALK | 0.0% | 61 | 1.5% | 395 | 0.336 |
| RNF43 | 0.0% | 61 | 0.5% | 395 | 0.580 |
| ERBB3 | 0.0% | 72 | 3.0% | 395 | 0.137 |
| NOTCH1 | 0.0% | 72 | 2.0% | 395 | 0.227 |
| ABL1 | 0.0% | 86 | 0.5% | 395 | 0.512 |
| AKT2 | 0.0% | 86 | 0.3% | 395 | 0.611 |
| BRAF | 0.0% | 97 | 0.0% | 395 | 1.000 |

**Table S5.** Patients' characteristics among patients who received a clinical recommendation (n=116) and patients with non-matched therapy (n=47).

|  | **Received recommendations (n=116)** | **Nonmatched therapy (n=47)** |
| --- | --- | --- |
| **Age (years)** | | |
| **Median (range)** | 54 (33-80) | 53 (29-80) |
| <50 | 41 (35%) | 16 (34%) |
| >= 50 | 75 (65%) | 31 (66%) |
| **ECOG** | | |
| 0 | 49 (43.0%) | 18 (38.3%) |
| 1 | 62 (54.4%) | 26 (55.3%) |
| 2 | 3 (2.6%) | 3 (6.4%) |
| **Hormone receptor status** | | |
| Negative | 11 (9.5%) | 17 (36.2%) |
| Positive | 105 (90.5%) | 30 (63.8%) |
| **HER2 status in primary tumor** | | |
| Negative | 102 (88%) | 40 (85.1%) |
| Positive | 14 (12%) | 7 (14.9%) |
| **Previous lines of metastatic treatment** | | |
| 1 | 27 (23.3%) | 12 (25.5%) |
| 2-3 | 30 (25.9%) | 13 (27.7%) |
| ≥3 | 59 (50.8%) | 19 (40.4%) |

**Table S6.** Clinical and directed therapy received recommendations according to the somatic mutations (N = 116).

| **Targeted therapies** | **N** |
| --- | --- |
| AKT inhibitors | 104 |
| Therapies targeting PI3K or AKT | 89 |
| PAN-ERBB tyrosine kinase inhibitor | 8 |
| Selective ER modulators (SERMs) and/or the selective ER degrader (SERD) | 8 |
| NOTCH inhibitors | 3 |
| FGFR1-4 tyrosine kinase inhibitor | 2 |
| CDK4/6 inhibitors | 2 |
| BRAF inhibitors | 1 |
